# Novel variants in PDE6A and PDE6B genes and its phenotypes in patients with retinitis pigmentosa in Chinese families

**DOI:** 10.1186/s12886-021-02242-5

**Published:** 2022-01-15

**Authors:** Yuyu Li, Ruyi Li, Hehua Dai, Genlin Li

**Affiliations:** Beijing Tongren Eye Center, Beijing Tongren Hospital, Capital Medical University, Beijing Ophthalmology & Visual Sciences Key Lab, Beijing, 100730 People’s Republic of China

**Keywords:** Retinitis pigmentosa, PDE6A,PDE6B, Novel variants, Phenotypes

## Abstract

**Background:**

Retinitis pigmentosa (RP) is a genetically heterogeneous disease with 89 causative genes identified to date. However, only approximately 60% of RP cases genetically solved to date, predicating that many novel disease-causing variants are yet to be identified. The purpose of this study is to identify novel variants in PDE6A and PDE6B genes and present its phenotypes in patients with retinitis pigmentosa in Chinese families.

**Methods:**

Five retinitis pigmentosa patients with PDE6A variants and three with PDE6B variants were identified through a hereditary eye disease enrichment panel (HEDEP), all patients’ medical and ophthalmic histories were collected, and ophthalmological examinations were performed, followed by an analysis of the possible causative variants. Sanger sequencing was used to verify the variants.

**Results:**

We identified 20 variants in eight patients: 16 of them were identified in either PDE6A or PDE6B in a compound heterozygous state. Additional four heterozygous variants were identified in the genes ADGRA3, CA4, OPTN, RHO. Two novel genetic changes in PDE6A were identified (c.1246G > A and c.1747 T > A), three novel genetic changes in PDE6B were identified (c.401 T > C, c.2293G > C and c.1610-1612del), out of the novel identified variants one was most probably non-pathogenic (c.2293G > C), all other novel variants are pathogenic. Additional variant was identified in CA4 and RHO, which can cause ADRP (c.243G > A, c.688G > A). In addition, a novel variant in ADGRA3 was identified (c.921-1G > A).

**Conclusions:**

This study reveals novel and known variants in PDE6A and PDE6B genes in Chinese families with autosomal recessive RP, and expands the clinical and genetic findings of photoreceptor-specific enzyme deficiencies.

## Background

Retinitis pigmentosa (RP, OMIM 268000) is a heterogeneous group of inherited retinal dystrophy (IRD) characterized by night blindness, retinal degeneration with bone spicule pigmentation, constricted visual fields, and progressive disease course. The prevalence of RP is approximately 1 per 4000 persons [1].

Retinitis pigmentosa (RP) is a genetically heterogeneous disease with 89 causative genes identified to date. However, only approximately 60% of RP cases genetically solved to date, predicating that many novel disease-causing variants are yet to be identified (https://sph.uth.edu/retnet/sum-dis.htm 2021.04.28). The gene therapy and stem cell therapy for retinitis pigmentosa has a promising future, so the identification of novel causative variants is becoming increasingly important.

Phosphodiesterase 6(PDE6) enzyme is a heterotetrameric protein consisting of alpha (PDE6A;180,071), beta (PDE6B; 180072), and 2 gamma subunits (PDE6G; 180,073) [2]. Both alpha and beta subunits are required for full phosphodiesterase activity, and mutations in genes encoding those subunits are known to cause autosomal recessive RP. The mechanisms by which PDE6A and PDE6B mutations lead to RP are probably similar because PDE6A and PDE6B subunits are enzymatically equivalent [3] and may lead to rod followed by cone death [4].

Mutations in PDE6A are found in a very low percentage of patients with RP as showed first in a study by Huang and coworkers, suggesting a frequency of < 1% [3]. Screening of about 160 patients with recessive RP in North America in a subsequent study found a frequency of mutations of approximately 3–4% [4]. Mutations in PDE6B are found in a frequency of about 4% in patients from North America [1, 5–7]. There is no statistics date about incidence rate in Chinese family. Because of the low incidence, many novel disease-causing variants are yet to be identified. The purpose of this study is to report the causative variants of Chinese RP families with PDE6A and PDE6B variants, expanding the clinical and genetic findings of photoreceptor-specific enzyme deficiencies.

## Materials and methods

### Ethics approval

All experiments involving patient DNA, as well as DNA from related individuals, were approved by the Clinical Research Ethics Committee in Beijing Tongren Hospital, Capital Medical University. The ethics committees approved this consent procedure (TREC2015-XJS07).

### Patients

Eight patients from eight unrelated families were enrolled in this retrospective study. We identified five RP patients with PDE6A variants and three with PDE6B variants. All patients were recruited from the Department of Ophthalmology, Beijing Tongren Eye Center. Clinical diagnosis of RP was made based on clinical evaluation and electroretinograms. All medical and surgical records for the patient were reviewed. The ophthalmic examinations performed in the study patient included decimal best-corrected visual acuity (BCVA), slit lamp, funduscopy, fundus photography, visual field testing, electroretinography (ERG), optical coherence tomography (OCT) and fluorescein angiography (FFA). One hundred Chinese Han healthy individuals were selected as the control group.

### Mutation screening by HEDEP

Blood samples were obtained from the patients, and genomic DNA was extracted by using standard protocols. A specific hereditary eye disease enrichment panel (HEDEP) based on targeted exome capture technology was used to collect the protein coding regions of 441 hereditary eye disease genes. Exon-enriched DNA libraries were then subjected to high-throughput sequencing using the Illumina HiSeq platform. Targeted gene enrichment, high-throughput sequencing, and data analysis were performed as described previously [8]. Briefly, exons of the target genes and adjacent portions of introns were captured by probe hybridization; enriched target genes were then sequenced with the Illumina HiSeq platform. Specific pathogenic mutations were verified by Sanger sequencing. Genetics company do analyze to the data and give us the results.

### Mutation validation by Sanger sequencing

Specific pathogenic mutations were verified by Sanger sequencing using four programs to evaluate the identified missense variants included mutation taster (MutationTaster), the PolyPhen2 (http://genetics.bwh.harvard.edu/pph2/), SIFT (http://sift.bii.a-star.edu.sg/index.html), and PROVEAN (http:// provean.jcvi.org/index.php) programs. BDGP (https://www.fruitfly.org/seq tools/splice.html), Netgene (http://www.cbs.dtu.dk/services/NetGene2/) were used to evaluate the identified splicing variants. Meanwhile, the frequency of the identified variants in controls was assessed using gnomAD. Pathogenicity of all mutations was evaluated following American College of Medical Genetics and Genomics (ACMG) criteria.

## Results

Eight patients from China were included in this study, 5 (62.5%) were male and 3 (37.5%) were female. The mean years was 36 (range,12-47 years). Probands P01 to P05 carried PDE6A variants while probands P06 to P08 carried PDE6B variants. All the identified variants were identified in a heterozygous state. A total of 20 different variants were identified, including 11 missense variants, one nonsense mutation, three splicing mutations, and one deletion (Table 1).

**Table 1:** variants identified in this study

Family	Gene	Nucleotide variant	Protein variant	Polyphen	Mutation Taster	SIFT	PROVEN	VF in gnomAD	Previously reported
P01	PDE6A	c.1349 T > C	p. Phe450Ser	Benign	Disease causing	Tolerated	Neutral	0.21%	Yes [9]
	PDE6A	c.1246G > A	p. Asp416Asn	Probably damaging	Disease causing	Deleterious	Deleterious	0%	No
	CA4	c.243G > A	p. Trp81*	NA	NA	NA	NA	0%	No
P02	PDE6A	c.1685G > A	p. Arg562Gln	Possibly damaging	Disease causing	Deleterious	Deleterious	0.0028%	Yes [10]
	PDE6A	c.1467 + 1G > C	p.?	NA	NA	NA	NA	0.0080%	Yes [11–13]
P03	PDE6A	c.2275-2A > G	p.?	NA	NA	NA	NA	0%	Yes [14]
	PDE6A	c.1957C > T	p. Arg653*	NA	NA	NA	NA	0.0028%	Yes [15]
P04	PDE6A	c.1747 T > A	p. Tyr583Asn	Possibly damaging	Disease causing	Tolerated	Deleterious	0%	No
	PDE6A	c.1651A > G	p. Lys551Glu	Benign	Disease causing	Deleterious	Deleterious	0%	Yes [10]
	OPTN	c.1634G > A	p. Arg545Gln	Benign	Disease causing	Tolerated	Neutral	0.3103%	Yes [16, 17]
P05	PDE6A	c.1651A > G	p. Lys551Glu	Benign	Disease causing	Deleterious	Deleterious	0%	Yes [10]
	PDE6A	c.285C > A	p. Ser95Arg	Possibly damaging	Disease causing	Deleterious	Deleterious	0%	Yes [10]
P06	PDE6B	c.401 T > C	p. Leu134Pro	Probably damaging	Disease causing	Deleterious	Deleterious	0.0037%	No
	PDE6B	c.2293G > C	p. Ala765Pro	Benign	Polymorphism	Deleterious	Neutra	0.04182%	No
P07	PDE6B	c.385G > A	p. Glu129Lys	Probably damaging	Disease causing	Deleterious	Deleterious	0.0014%	Yes [18]
	PDE6B	c.1610-1612del	p. 537-538del	NA	NA	NA	NA	0%	No
P08	PDE6B	c.1467 + 1G > C	p.?	NA	NA	NA	NA	0.0008%	Yes [19]
	PDE6B	c.2204 T > C	p. Leu735Pro	Probably damaging	Disease causing	Deleterious	Deleterious	0.0004%	Yes [10]
	RHO	c.688G > A	p. Val230Ile	Probably damaging	Disease causing	Tolerated	Neutral	0.0039%	No
	ADGRA3	c.921-1G > A	p.?	NA	NA	NA	NA	NA	No

*VF in gnomAD*: the variants frequency in health population in gnomAD; *NA*: data not available

We identified two novel variants in PDE6A (c.1246G > A and c.1747 T > A), three novel genetic changes in PDE6B (c.401 T > C, c.2293G > C and c.1610-1612del), an additional novel variants were identified in CA4 (c.243G > A) and RHO (c.688G > A) genes. Out of the novel identified variants one was most probably non-pathogenic (c.2293G > C), additional variants had conflicting interpretations of pathogenicity.

The mean (SD) BCVA was 0.93 (0.92) logMAR (range, 0.1 to 2.30; 16 eyes). The clinical date was present in Table 2.

**Table 2:** Clinical findings in 8 patients

Sings and symptoms	P01	P02	P03	P04	P05	P06	P07	P08
Gender	male	male	female	female	male	female	male	female
Age (year)	12	28	34	36	47	42	42	47
Nyctalopia time	First decade	First decade	First decade	First decade	First decade	First decade	First decade	First decade
Course of disease (year)	5	25	30	30	40	35	35	40
BCVA (logMAR) OD	0.4	0.1	0.5	0.2	HM	1/35 at 1 m	0.4	HM
BCVA (logMAR) OS	0.5	0.1	0.5	0.3	HM	0.4	0.4	HM
bone-spicule pigmentation	–	–	+	+	+	+	+	+
ERM	–	+	+	+	+	+	–	+
CME	–	–	+	–	–	–	–	–
Macular atrophy	–	–	+	–	+	+	+	+
PSAWM	–	–	+	–	–	–	–	–
Lamellar macular hole	–	–	+	–	–	–	–	–
CST (um) OD	296	224	169	229	419	138	181	NA
CST (um) OS	NA	229	366	215	193	134	221	NA

*Abbreviations*: - = feature not present, + = feature present, *OD* Right eye, *OS* Left eye, *CME* Cystoid macular edema, *ERM* Epiretinal membrane, *PSAWM* Posterior staphyloma associated with myopia, *CST* Central subfield thickness; BCVA (at present age)

**Fig. 1:**
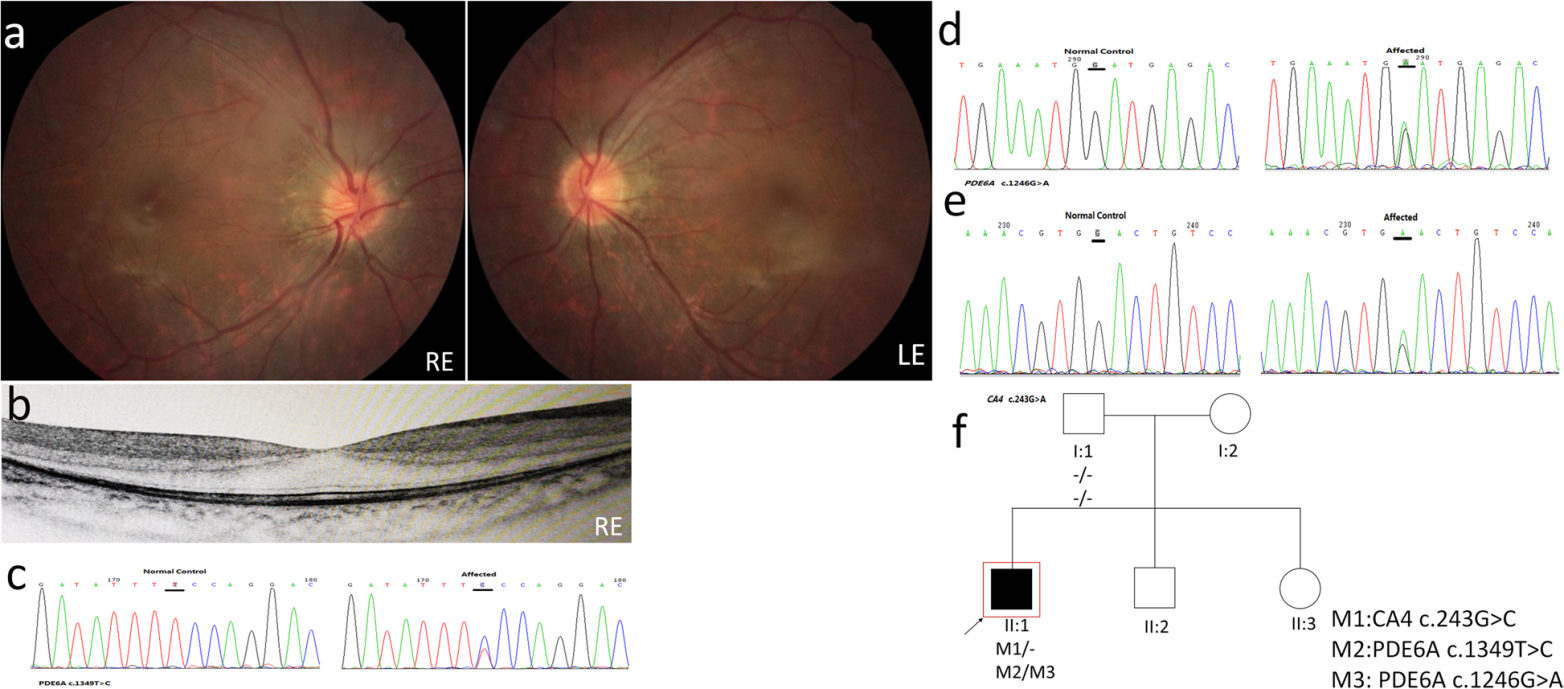
Clinical observations and identification of variants in P01

Patient P01 is a 12-year-old male. Night blindness was the first symptom noted at the age of 6 years old. Fundus images show relatively mild retinal degeneration, swelling of the nerve fiber layer causes unclear optic disc boundaries and tortuous venous of both eyes (Fig. 1a). Central macular thickness was 296 μm in the right eye (Fig. 1b). (OCT scan was not available for the left eye). Two novel variants were identified in this index case, one in PDE6A (c.1246G > A) and one in CA4 (c.243G > A) (Fig. 1c-e). Both are damaging according to all online prediction programs. The nonsense mutation in CA4: c.243G > A leads to premature termination of protein translation and can cause autosomal dominant hereditary retinitis pigmentosa, it is probably pathogenic and affects the phenotype of P01. Those variants were not found in the gnomAD database, and hence we believe that the variant in CA4 is pathogenic and cause RP in this proband. Genotyping of proband’s father revealed no mutations, indicating that both variants in PDE6A are probably on the same allele (Fig. 1f). Mutations in PDE6A cause an autosomal recessive RP and both alleles should carry mutations, so it is probably not the causative gene.

**Fig. 2:**
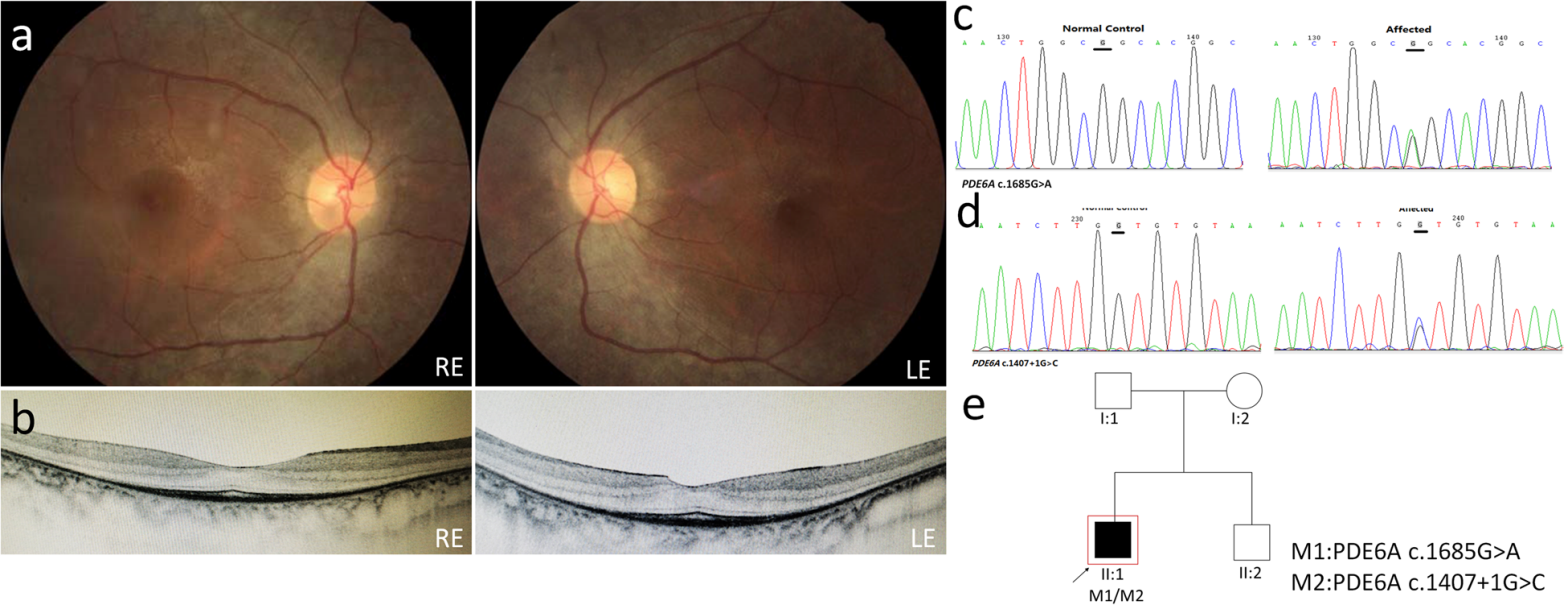
Clinical observations and identification of variants in P02

Proband P02 is a 28-year-old male. Fundus examination shows moderate retinal degeneration and retinal arteriolar attenuation (Fig. 2a). OCT images of P02 show nearly normal thickness of macula, mild epiretinal membrane mainly in the macular area, conserved IS/OS lines, shorter than presented in the normal fundus (Fig. 2b).

We identified two known mutations in PDE6A (c.1685G > A and c.1407 + 1G > C) in the index case (Fig. 2c, d), but weren’t able to check segregation because no other family member was genotyped (Fig. 2e).

**Fig. 3:**
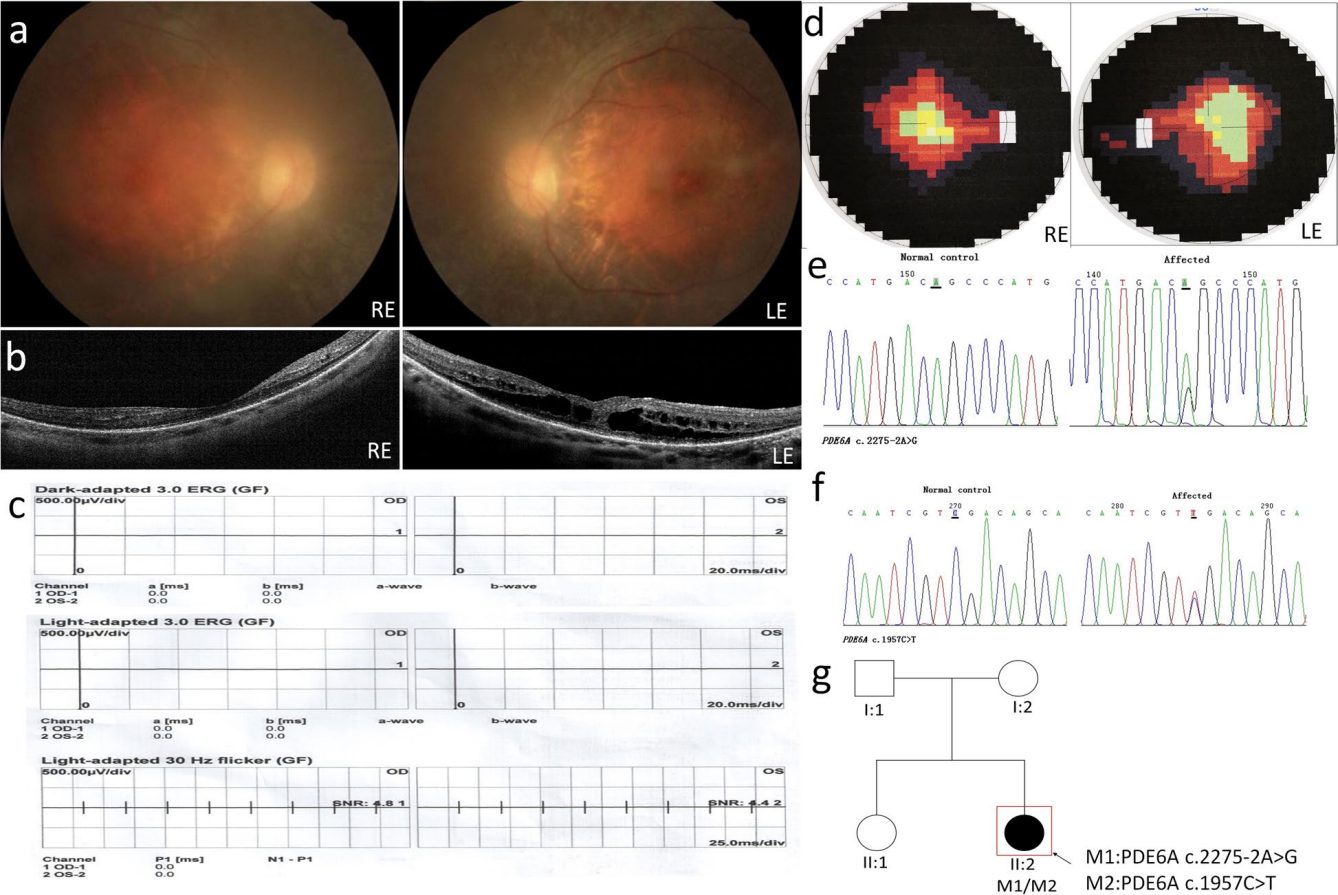
Clinical observations and identification of variants in P03

The index case P03 is a 34-year-old female. She had cataract surgery in both eyes at the age of 25 due to posterior capsular opacity. Fundus images show macular atrophy and disruption of the entire ellipsoid zone in the right eye (Fig. 3a). Epiretinal membrane, cystoid macular edema, outer retinoschisis and lamellar macular hole in the left eye are presented in OCT scans (Fig. 3b) and accompanied by very high myopia (OD: -10.50DS/+ 2.00 DC × 90°, OS: -9.50DS/+ 1.25 DC × 75°) that might aggravate other symptoms of the disease. ERG responses to all stimuli were not detectable (Fig. 3c). Visual fields were severely constricted to 10°at the age of 15 (Fig. 3d). We identified two known mutations in PDE6A in a heterozygous state (c.2275-2A > G and c.1957C > T) (Fig. 3e, f) in that patient but weren’t able to check segregation because no other family member was genotyped (Fig. 3g).

**Fig. 4:**
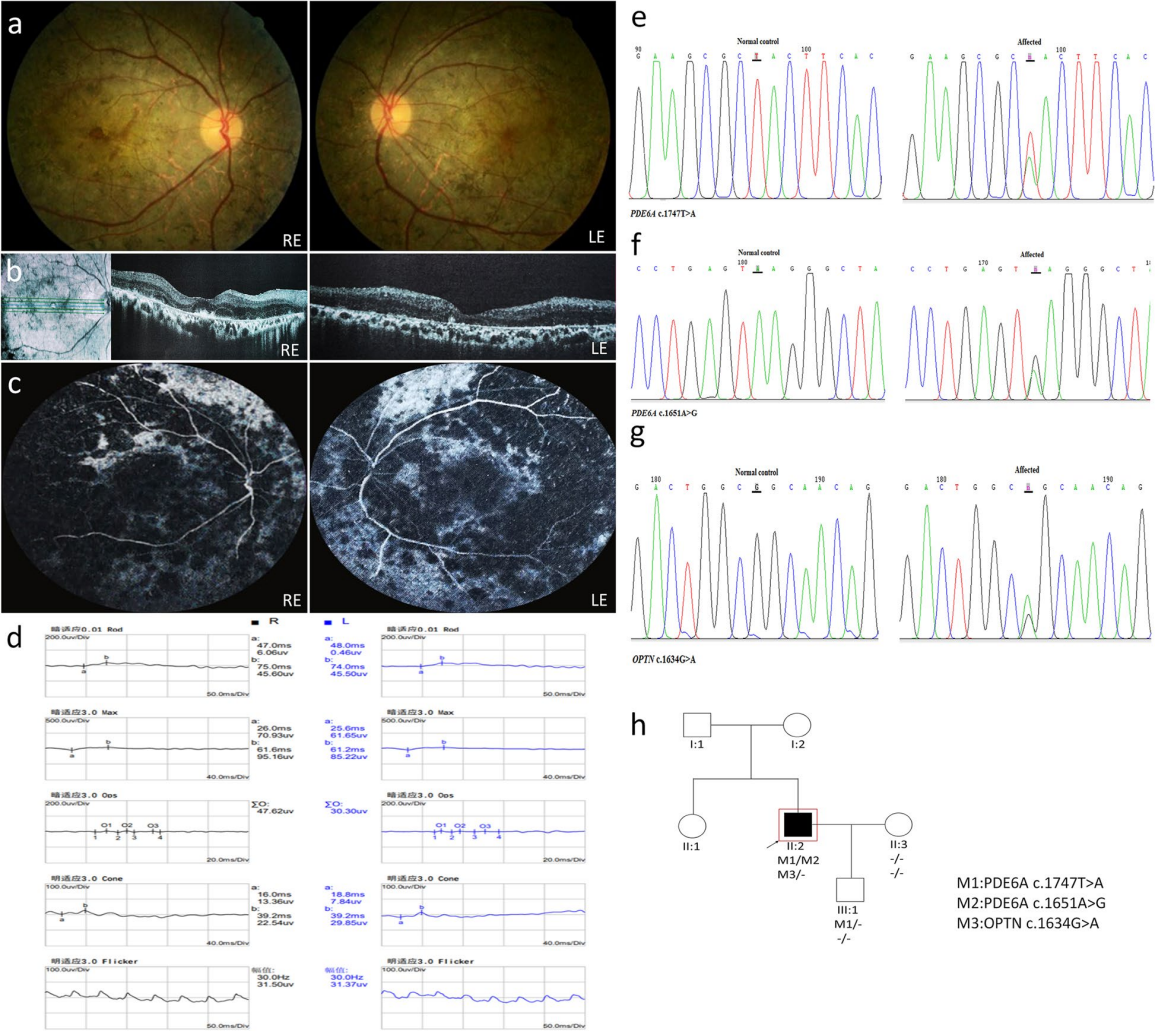
Clinical observations and identification of variants in P04

Patient P04 is a 36-year-old male. Fundus images show extensive intraretinal pigment migrations extending from the mid-periphery equatorial region to the arcades in both eyes with extensive arterial attenuation, macular and peripapillary atrophy, only central 1 PD foveal island was sparing (Fig. 4a). OCT images show high-density deposits on the surface of RPE layer in macula, residual intraretinal vacuoles and an entirely disrupted and atrophy of the retina and macula, the outer retinal structures are lost (Fig. 4b). Fluorescein angiography show heterogeneous hyperautofluorescence with hypoautofluorescent fovea compatible with retinal atrophy (Fig. 4c). The full-field ERG shows a decrease in rod and cone amplitudes in rod response and combined rod-cone response, as well as a delayed implicit time. The 30 Hz Flicker cone response also shows a decreased amplitude (Fig. 4d). Several variants were identified in patient P04: one known variant in OPTN (c.1634G > A) that was previously reported as benign, and two additional variants in PDE6A (a novel c.1747 T > A variant and a known c.1651A > G variant) (Fig. 4e-g). The novel variant was predicted by most of the online prediction programs as damaging (Table 1) and was not reported previously in the gnomAD database. The two PDE6A variants are located on different alleles, as can be concluded from the genotype of III1 (Fig. 4h). Those findings indicate that the variants identified in PDE6A are the cause of the disease.

**Fig. 5:**
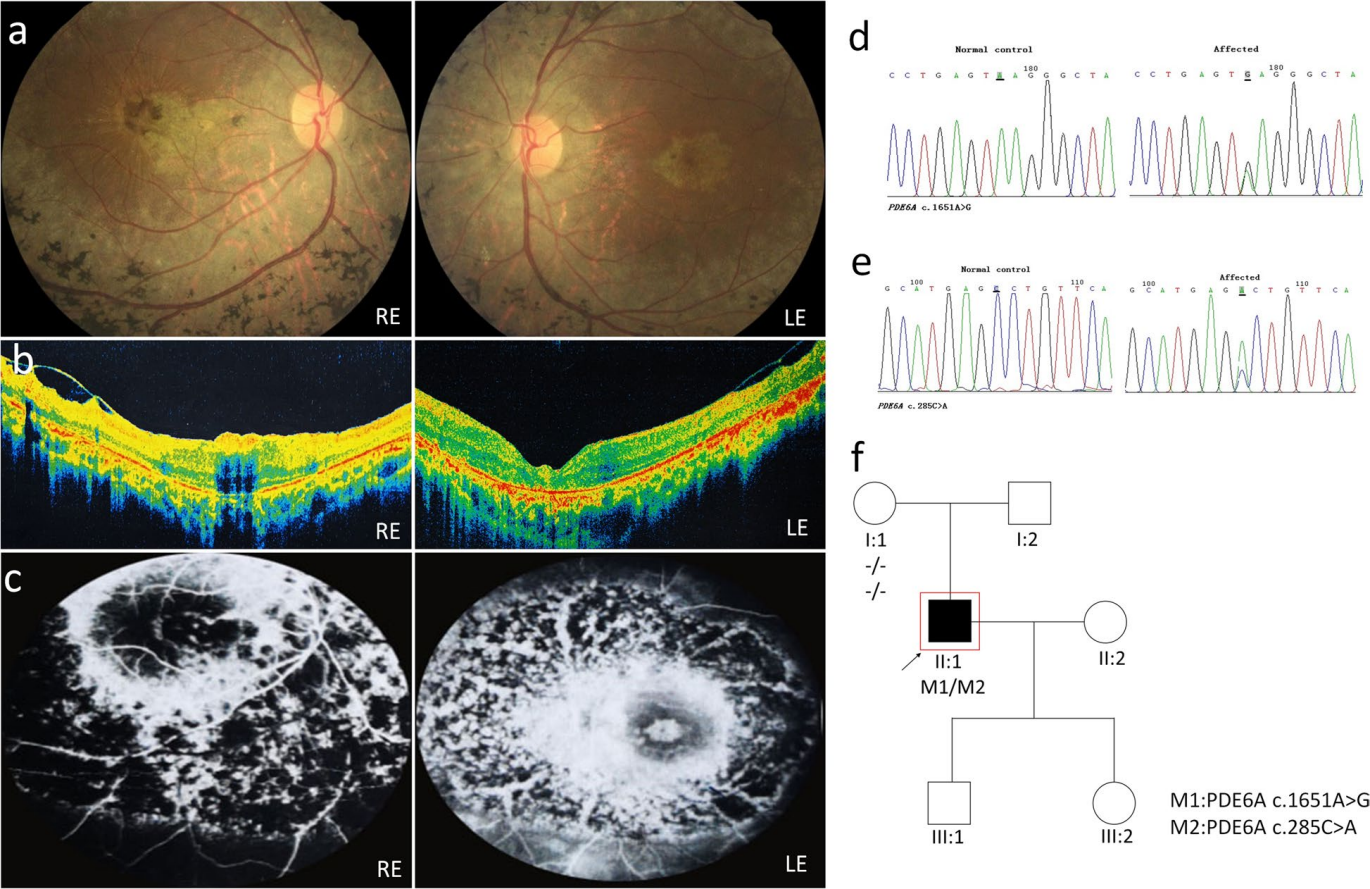
Clinical observations and identification of variants in P05

Proband P05 is a 47-year-old male. Fundus images show spread retinal degeneration with severe chorioretinal atrophy and bone spicule pigmentation mainly in the periphery, but also in the macular area (Fig. 5a). Those changes identified in fundoscopy are compatible with macular atrophy and structure change identified in OCT. OCT images show epiretinal membrane in the macular area, which caused vitreomacular traction. Disruption of the ellipsoid zone in both eyes (Fig. 5b). Fluorescein angiography revealed heterogeneous hyperautofluorescent spots in the whole retina. Hyperautofluorescent areas are more notable in the central retina, and in the periphery, a combination of hyperautofluorescent, as well as hypoautofluorescent spots is notable (Fig. 5c). We identified two known mutations in PDE6A in a heterozygous state (c.1651A > G and c.285C > A) (Fig. 5d, e) in that patient but weren’t able to check segregation because no other family member was genotyped (Fig. 5f).

**Fig. 6:**
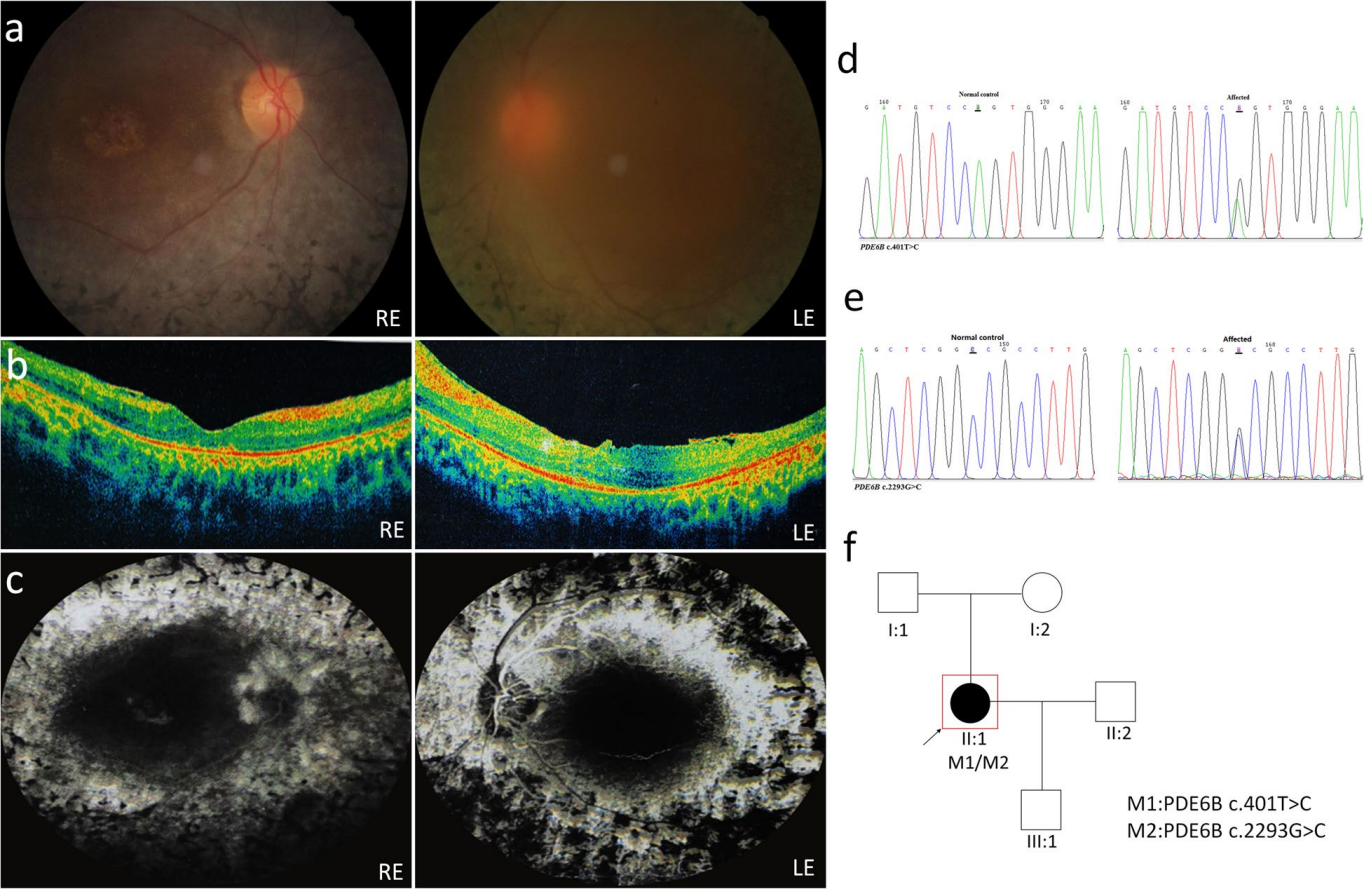
Clinical observations and identification of variants in P06

Patient P06 is a 42-year-old female. Anterior segment examination show severe subcapsular cataract in left eye which led to unclear fundus images in this eye. Ophthalmoscopy showed attenuated vessels, and mid-peripheral bone-spicule pigmentation (Fig. 6a). Significant macular atrophy and exudates in outer plexus layer can be seen in both eyes, with more severe appearance in LE. Epiretinal membrane was identified in both eyes: mainly in the macular area in RE and extensive epiretinal membranes with thickened hyaloid were identified in LE. Thinning of the outer nuclear layer (ONL) and disruption of the ellipsoid zone (EZ) and external limiting membrane (ELM) can be seen in both eyes (Fig. 6b). Fluorescein angiography show heterogeneous hyperautofluorescent areas in the periphery and hypoautofluorescent fovea. The hyperautofluorescent spots clearly demarcate the atrophic areas (Fig. 6c). Phenotypic differences between the two eyes illustrate that macular atrophy may significant affect vision than extensive epiretinal membranes. Two variants were identified in PDE6B gene in this index case (c.401 T > C and c.2293G > C) (Fig. 6d, e), both are novel. The first novel variant was predicted by all the online prediction programs as damaging, its frequency in gnomAD was very low (0.0037%) and thus it is probably a pathogenic variant. In contrast, the second variant was predicted by all the online prediction programs as non-pathogenic, its frequency in gnomAD was much higher (0.04182%). No other family member was genotyped (Fig. 6f). Based on those findings we concluded that this variant is not pathogenic and the disease in this patient does not cause by PDE6B.

**Fig. 7:**
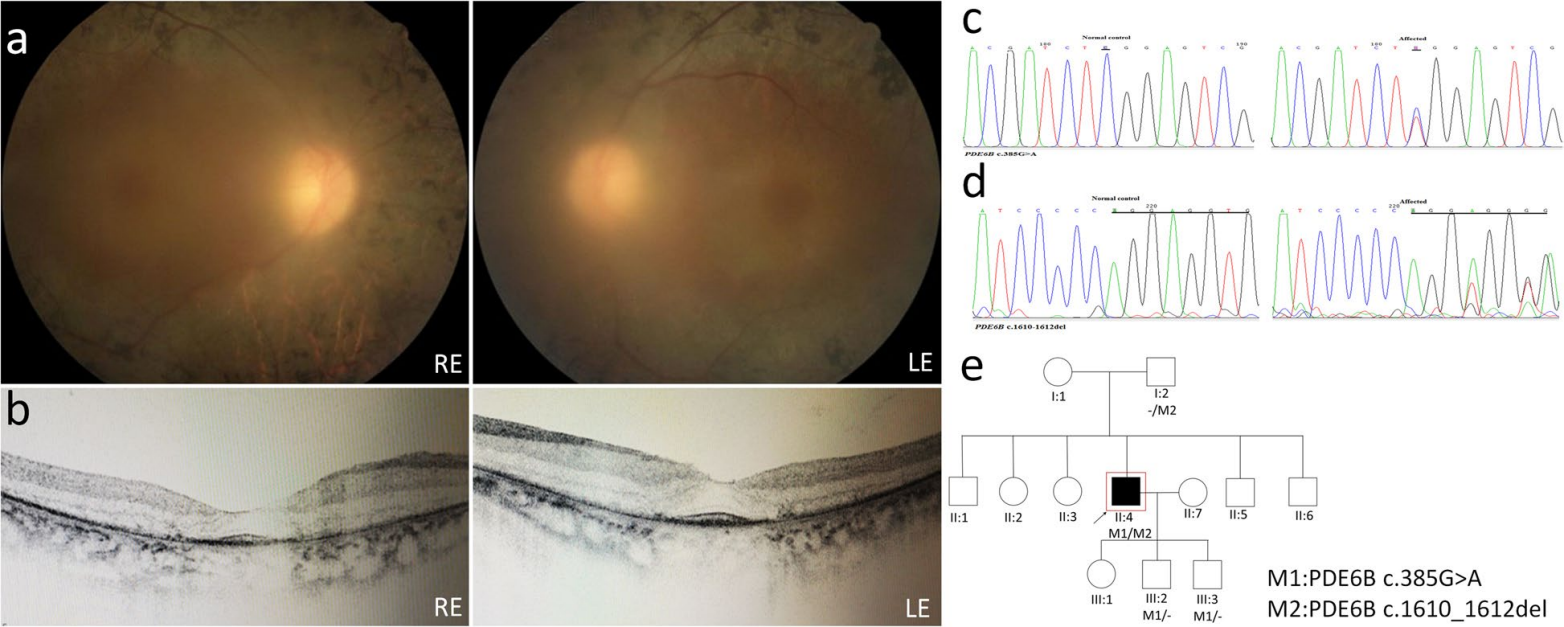
Clinical observations and identification of variants in P07

Patient P07 is a 42-year-old male. Anterior segment examination shows posterior subcapsular cataracts in both eyes which was the main cause for blurred and not clear fundus images. Attenuated vessels and mid-peripheral bone-spicule pigmentation were the main observations in fundus images (Fig. 7a). On OCT residual ONL and an intact EZ was seen in the foveal area in both eyes with thinning and loss of ONL in para-foveal areas (Fig. 7b). Two variants were identified in PDE6B gene in this index case (c.385G > A and c.1610-1612del) (Fig. 7c, d), one is novel (c.1610-1612del). Variant c.1610-1612del in PDE6B causes shifting of all codons after code1610, so it causes an inframe deletion and affect protein structure and function. This variant was not identified in the gnomAD database, indicating that it is a very rare variant. Other family members were checked for those mutations (Fig. 4d). The mother of the index case was identified as a carrier for c.385G > A, and two sons of the index case were identified as carriers for c.1610-1612del, which indicates that those variants are located on different alleles (Fig. 7e). We assume that this variant, together with the previously reported c.385G > A variant, causes RP in this patient.

**Fig. 8:**
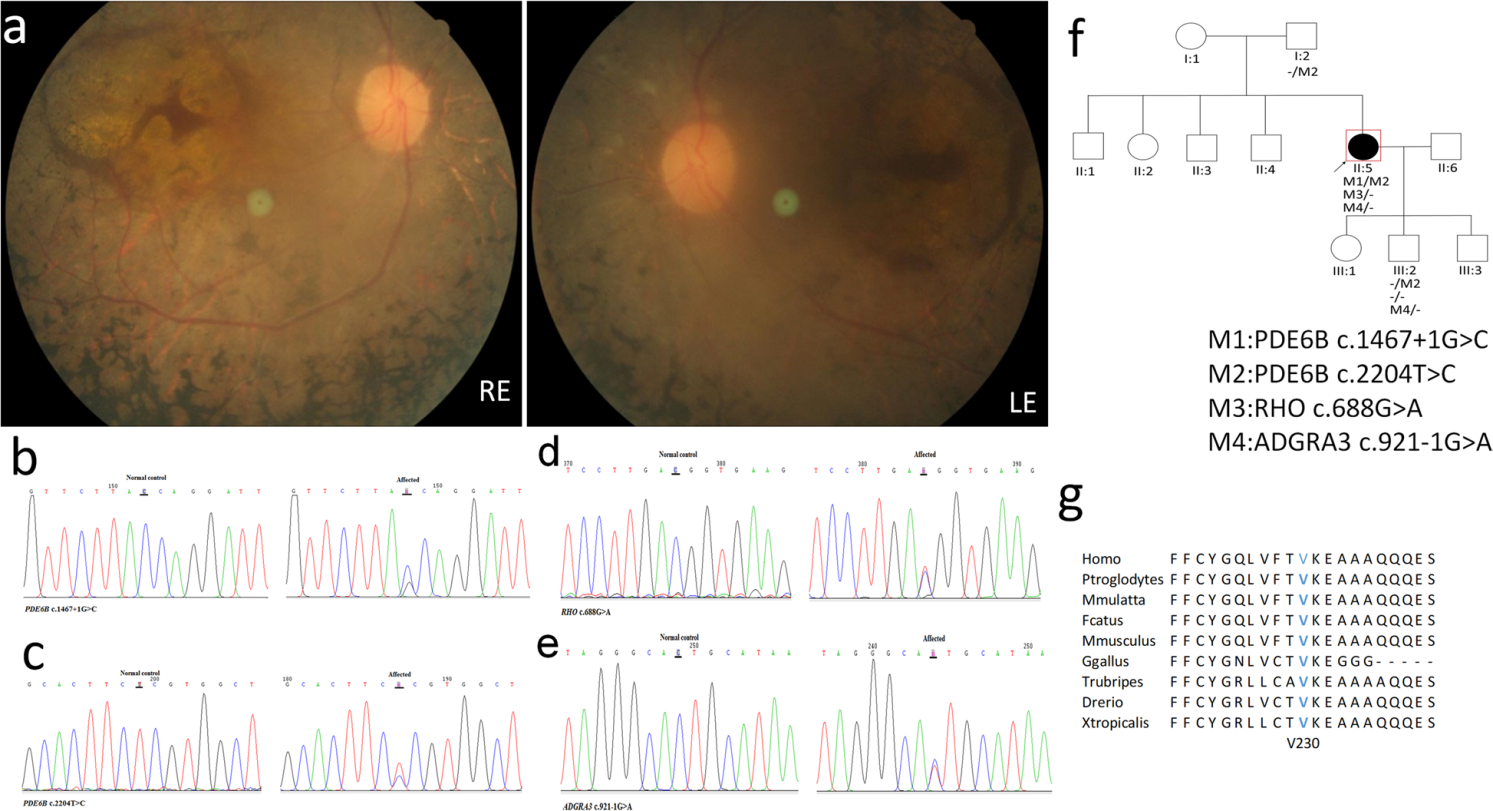
Clinical observations and identification of variants in P08

Patient P08 is a 47-year-old female. Fundus images show macular atrophy and peripapillary atrophy, attenuated vessels, and mid-peripheral bone-spicule pigmentation (Fig. 8a). The index case (p08) was identified with four possible variants which can affect her vision, two of them in PDE6B, one in RHO and one in ADGRA3 (Fig. 8b-e). The parents and four siblings of the index case are healthy, as well as her three children. One of the children was genetically examined and identified as heterozygous for two variants c.2204 T > C in PDE6B and c.921-1G > A in ADGRA3 (Fig. 8f), indicating that (a) the two mutations identified in the index case in PDE6B are located on different alleles and are probably the main cause of her disease and (b) a heterozygous mutation in ADGRA3 is not pathogenic. Additional novel variant (c.688G > A) was identified in the RHO gene which is known to cause autosomal dominant RP. Even though this missense variant affects a conserved amino acid (we compare this region to other species then found the affected amino acid is conserved among other species) and suspected to affect protein structure and function (Fig. 8g), we are still not sure about its pathogenicity. Online prediction programs are controversial regarding this variant, and only full segregation analysis in the family can unravel the true nature of this variant. A novel splicing variant in ADGRA3 was identified in a heterozygous state, while ADGRA3 is known to cause autosomal recessive retinitis pigmentosa, and thus can not be disease-causing in this state.

## Discussion

The phosphodiesterase 6 enzyme is involved in hydrolysis of cGMP in the photoreceptors during the transduction of light signals. This enzyme is a heterotetrameric protein and it consists of alpha, beta and 2 gamma subunits. Both alpha and beta subunits are required for full phosphodiesterase activity [3]. Mutations in PDE6B were reported previously to cause autosomal dominant congenital stationary night blindness or autosomal recessive retinitis pigmentosa, while mutations in PDE6A were reported to cause retinitis pigmentosa that is inherited in an autosomal recessive manner only (OMIM). The mutation of PDE6A causes retinitis pigmentosa 43, which affects the function of PDE6B [20]. Phenotypic analysis revealed no substantial differences between the two groups except for night blindness as a symptom that was noted to be more prevalent in the PDE6A than PDE6B group by another group [21].

We identified five RP patients with PDE6A variants and three with PDE6B variants, all our patients reported night blindness as the first sign appeared from birth. It seems that this sign is one of the most prominent feature of RP due to PDE6A or PDE6B mutations, as it was reported previously by many other groups as a first sign noted in those patients [7, 22–26]. ERG results were available only for 2 out of 8 patients, and were completely absent or severely reduced. Those results are compatible with ERG results of PDE6A or PDE6B patients that were reported in other researches [7, 22, 24, 25, 27]. OCT images showed a major reduction in the ONL and the EZ width, indicating the progression of the disease. Similar results were previously shown by others [26, 28, 29]. In 8 patients of different ages, at the age of 12 we can still observe some ONL and the EZ looks almost normal. But later, after the age of 30 the progression is very fast, ONL is barely noticed or totally absent, and there is a major constriction of the EZ among all patients, suggesting that PDE6A and PDE6B genes variants is a typical rod cell damage RP, secondary cone cell apoptosis occurs when course over 30 years. Several complications of macula due to PDE6A or PDE6B mutations were noted in our patients, mainly in older ages. CME was present in 6.25%, less than studies in PDE6A (25%) and PDE6B (35%) by Kuehlewein but similar to that in the general population of patients with RP [27, 28, 30–32]. ERM in 56% (50% in PDE6A and 66.6% in PDE6B), similar to Kuehlewein’s study about PDE6B (67%), higher than Kuehlewein’s study about PDE6A. So, the incidence of ERM in PDE6B may higher than PDE6A and general population of patients with RP. The frequency of macular atrophy is much higher than Kuehlewein’s study about PDE6A and PDE6B, this can much explain why our patients had much poor mean BCVA than other studies. Incidence of retinoschisis and lamellar macular hole is 6.25% (1/16 eyes), this patients’ RP was accompanied by high myopia, which can explain the abnormal splitting of the retina and the macular hole [33, 34].

In P01, swelling of the nerve fiber layer causes unclear optic disc boundaries and tortuous veins in both eyes. In addition, the macular fovea seems to be shallower in this patient. All those parameters differ him from other patients. P01 carries two PDE6A variants, one of which is predicted to be non-pathogenic and it is not clear whether those variants are located on different alleles. An additional variant was identified in this patient in the CA4 gene, which is responsible for AD RP. The mutation in CA4 is nonsense and leads to premature termination of CA4 protein translation, indicating that this mutation has a higher probability of being the cause of the disease. A definite answer to the question of what mutation or mutations cause the disease can be given only after genetic testing of the mother and the siblings of the index case.

In the index case P04, we identified two causative mutations in PDE6A and an additional variant in OPTN. This gene was previously reported to cause autosomal dominant open-angle glaucoma [18, 19], but the specific variant that was identified in P04, was already reported previously as non-pathogenic in two other articles, therefore it could not be pathogenic.

In P06, we identified two heterozygous novel variants in PDE6B: c.401 T > C (which was most probably pathogenic due to low frequency in gnomAD and positive predictions in all prediction programs) and c.2293G > C (which was most probably non-pathogenic due to high frequency in gnomAD and negative predictions in all prediction programs). In addition, we weren’t able to genotype other family members and prove that those two variants are located on different alleles. It is possible that the cause of RP in this patient is PDE6B, and the second mutation on the second allele was not identified by us due to several possible genetic events [35], 1) larger deletions or rearrangements that are not detectable by Sanger sequencing; (2) deep intronic mutations, which caused aberrant splicing, but were not examined in our study and (3) mutations in regulatory regions, which were not examined in our study. Because the phenotype of the patient does not differ from phenotypes of other PDE6A/B patients in this study, we believe that the second mutation was missed. But it is also possible that there might be additional mutations in other genes that are responsible for her RP.

Two known mutations in PDE6B were identified in P08 and the segregation test for those mutations showed clearly that they are located on different alleles and therefore are the cause for RP of this patient. Additional two novel variants in RHO and ADGRA3 were identified, and we tried to estimate their possible pathogenicity. Mutations in RHO can cause ADRP, which means that a single mutation in one allele can be the cause of the disease. The variant that was identified by us in RHO is missense (c.688G > A) and was identified in a heterozygous state. It is a very rare variant, but the predictions about its pathogenicity are controversial. Identification of this variant in other healthy family members could help us decide, but segregation analysis for this change was not performed because we didn’t obtain blood samples from other healthy family members. Although the affected amino acid is conserved among other species. The RHO mutation of the index most probably from his mother, but the mother isn’t affected, so, although the affected amino acid is conserved among other species, we still consider that this variant is not pathogenic. Mutations in ADGRA3 were reported previously to cause ARRP, and therefore we believe that a single heterozygous change in ADGRA3 in P08 is not the cause for RP in this patient. Though it can also be possible that due to the disadvantages of the method we used, we were not able to identify the second mutation.

Our study has several limitations due to methods that were used in genetic and clinical analyses. Follow-up of VA, OCT and fundus photos, ERG, VFs, refraction, and different complications of the disease, might give us a more complete picture of the course of the disease. Fundus photos that were taken, included only the posterior pole, the periphery fundus was not presented well because of equipment disadvantage. We believe that eight cases are a relatively small group of patients, and it is impossible to draw unequivocal conclusions about disease progression from such a small cohort. In addition, a more substantial segregation analysis in each family will give us more accurate results regarding the probability of a certain variant being pathogenic. Overall, this study reveals novel and known mutations in Chinese families with ARRP due to mutations in PDE6A and PDE6B. Those findings expand the clinical and genetic findings of photoreceptor-specific enzyme deficiencies.

## Conclusions

In conclusion, we identified two novel variants in PDE6A, three novel variants in PDE6B, one novel variant in CA4 and one novel variant in RHO. Among them, one of the variants in PDE6B is clearly non-pathogenic (c.2293G > C) and an additional variant identified in (c.688G > A) has conflicting interpretations of pathogenicity. All other novel variants are pathogenic. This study expanding the clinical and genetic findings in ARRP patients due to PDE6A or PDE6B mutations.

## Data Availability

The datasets generated and/or analysed during the current study are not publicly available due our agency don’t recommend us to deposit the raw genetic resource date public, but are available from the corresponding author on reasonable request.

## Abbreviations

RP: Retinitis Pigmentosa
PDE6A: Phosphodiesterase 6A
PDE6B: Phosphodiesterase 6B
BCVA: Best-corrected visual acuity
ERG: Electroretinography
OCT: Optical coherence tomography
FFA: Fluorescein angiography
HEDEP: Hereditary eye disease enrichment panel
IRD: Inherited retinal dystrophy
RE: Right eye
LE: Left eye
